# SKAP2 Modular Organization Differently Recognizes SRC Kinases Depending on Their Activation Status and Localization

**DOI:** 10.1016/j.mcpro.2022.100451

**Published:** 2022-11-21

**Authors:** Laurine Levillayer, Patricia Cassonnet, Marion Declercq, Mélanie Dos Santos, Louis Lebreton, Katerina Danezi, Caroline Demeret, Anavaj Sakuntabhai, Yves Jacob, Jean-François Bureau

**Affiliations:** 1 Unité de Génétique Fonctionnelle des Maladies Infectieuses (GFMI), CNRS UMR 2000, Institut Pasteur, Université de Paris, Paris, France; 2 Unité de Génétique Moléculaire des Virus à ARN (GMVR), CNRS UMR3569, Institut Pasteur, Université de Paris, Paris, France

**Keywords:** SKAP2, SRC kinase family, protein-protein interaction, modular architecture, luciferase complementation assay, mutagenesis, BLK, B Lymphocyte Kinase, DIM, domain of dimerization, DIMPH, dimerization and Pleckstrin homology domains, FGR, Feline Gardner-Rasheed sarcoma viral homolog, FRK, Fyn Related src family tyrosine Kinase, FYN, FGR-YES1-related Novel protein, HCK, Hematopoietic Cell Kinase, LCK, Lymphocyte-Specific Kinase, LYN, LCK/Yes-related Novel protein tyrosine kinase, NLR, Normalized Luciferase Ratio, PH domain, Pleckstrin Homology domain, SH2 domain, Src Homology 2 domain, SH3, domain Src Homology 3 domain, SKAP1, SRC Kinase Adaptor Phosphoprotein 1, SKAP2, SRC Kinase Adaptor Phosphoprotein 2, SRMS, Src-Related kinase lacking C-terminal regulatory tyrosine and N-terminal Myristylation Sites

## Abstract

Dimerization of SRC kinase adaptor phosphoprotein 2 (SKAP2) induces an increase of binding for most SRC kinases suggesting a fine-tuning with transphosphorylation for kinase activation. This work addresses the molecular basis of SKAP2-mediated SRC kinase regulation through the lens of their interaction capacities. By combining a luciferase complementation assay and extensive site-directed mutagenesis, we demonstrated that SKAP2 interacts with SRC kinases through a modular organization depending both on their phosphorylation-dependent activation and subcellular localization. SKAP2 contains three interacting modules consisting in the dimerization domain, the SRC homology 3 (SH3) domain, and the second interdomain located between the Pleckstrin homology and the SH3 domains. Functionally, the dimerization domain is necessary and sufficient to bind to most activated and myristyl SRC kinases. In contrast, the three modules are necessary to bind SRC kinases at their steady state. The Pleckstrin homology and SH3 domains of SKAP2 as well as tyrosines located in the interdomains modulate these interactions. Analysis of mutants of the SRC kinase family member hematopoietic cell kinase supports this model and shows the role of two residues, Y390 and K7, on its degradation following activation. In this article, we show that a modular architecture of SKAP2 drives its interaction with SRC kinases, with the binding capacity of each module depending on both their localization and phosphorylation state activation. This work opens new perspectives on the molecular mechanisms of SRC kinases activation, which could have significant therapeutic impact.

## Highlights

• Characterization of the SKAP2 modular organization recognizing SRC kinases.
• Each module is affected by the activation and/or the localization of SRC kinases.
• Subtle nonstudied differences in SKAP2 binding between SRC kinases exist.
• Mutations in hematopoietic cell kinase induce its degradation by the proteasome.

## Graphical Abstract

**Figure:**
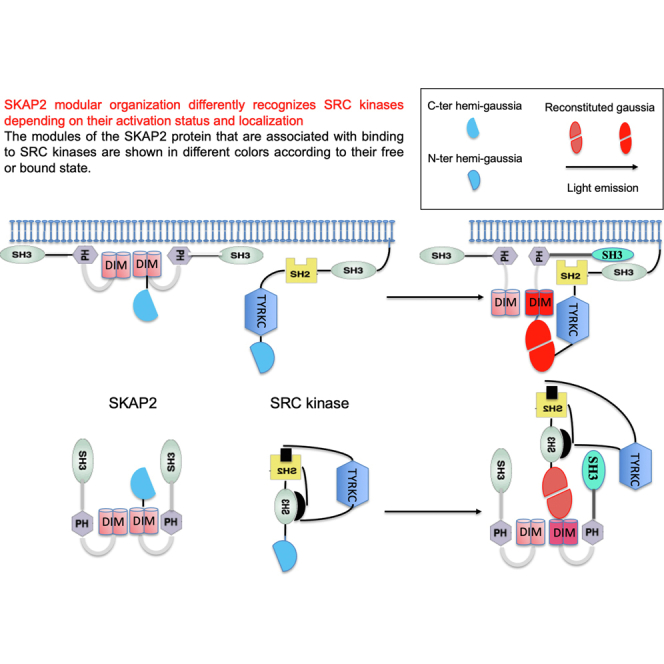

## In Brief

SRC kinases play multiple functions in different metabolic pathways. To perform each of these functions, they interact specifically with other proteins. This work describes how the SKAP2 protein, an assembly platform, interacts with these kinases to better understand their combined action. New and more specific therapies could be developed from this type of work, particularly in oncology.

**Fig. 1:**
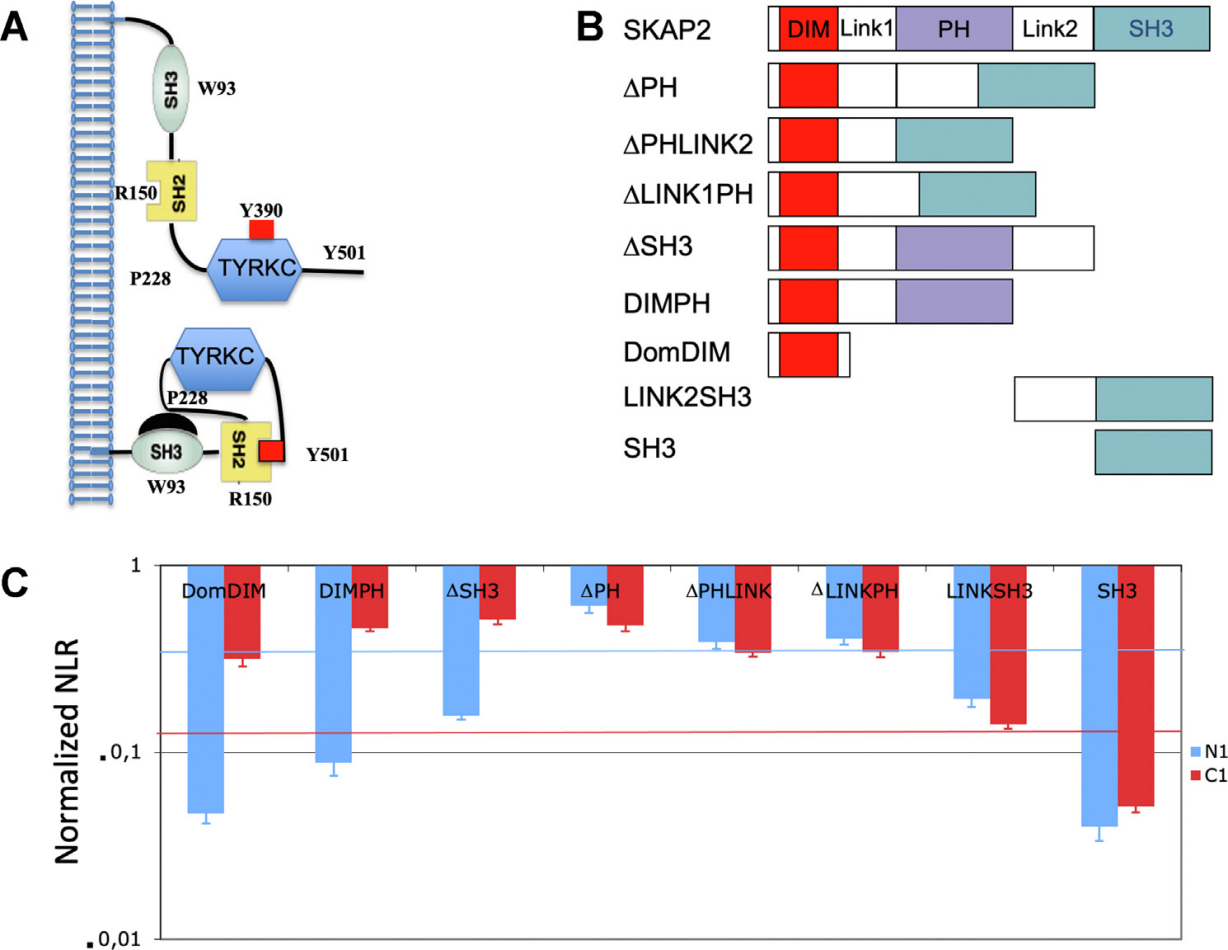
**Study of the interaction between SKAP2 deletion mutants and either N1-fused or C1-fused HCK.***A*, schematic illustrating inactive (*bottom*) and active (*top*) forms of HCK. *B*, schematic of SKAP2 deletion mutants. *C*, normalized NLR of the eight SKAP2 deletion mutants with N1-fused (*blue*) and C1-fused (*red*) HCK in a logarithmic scale. Normalized NLR is the ratio of the NLR between a N2-SKAP2 deletion mutant and one N1- or C1-SRC kinase to the corresponding NLR between wildtype N2-SKAP2 and the same Gluc1 SRC kinase. DIM, domain of dimerization; DIMPH, dimerization and Pleckstrin homology domains; HCK, hematopoietic cell kinase; NLR, normalized luminescence ratio; PH domain, Pleckstrin homology domain; SH3, Src homology 3 domain; SKAP2, SRC kinase adaptor phosphoprotein 2.

The SRC kinase family is composed of the SrcA subfamily with Feline Gardner-Rasheed sarcoma viral homolog (FGR), FGR-YES1–related novel protein (FYN), SRC, and YES, the SrcB subfamily with B lymphocyte kinase (BLK), hematopoietic cell kinase (HCK), lymphocyte-specific kinase (LCK), and LCK/Yes-related novel protein tyrosine kinase (LYN), and finally Fyn-related Src family tyrosine Kinase (FRK) (1). An SRC-related kinase, Src-related kinase lacking C-terminal regulatory tyrosine and N-terminal myristylation sites (SRMS), also exists lacking a C-terminal tyrosine which phosphorylation status regulates kinase activity and N-terminal myristylation sites. SRC kinases phosphorylate tyrosine residues and are composed of three domains, a SRC homology 3 (SH3) domain, a SRC homology 2 (SH2) domain, and a tyrosine kinase catalytic domain (supplemental Fig. S1*B*). SRC kinase activity is modulated by a fine allosteric control that modulates the passage from closed to open conformations (2, 3, 4). SRC kinases adopt either a closed, inactive conformation provided by intramolecular interactions between (i) the SH3 domain and a proline-rich motif located between the SH2 and kinase domains and (ii) the SH2 domain and a phosphorylated tyrosine motif in the C terminus of the protein or an open active conformation without these intramolecular interactions (Fig. 1*A*). The active conformation is locked by transphosphorylation of a tyrosine positioned in the active loop of the catalytic site (5). This phosphorylation also induces the appearance of a SH2 binding motif for Cbl ubiquitin ligases that controls SRC kinase degradation (6). The localization of most SRC kinases in different membranous subcellular compartments is finely regulated by N-terminal myristylation and palmitoylation with some differences between each SRC kinase (7, 8). However, neither SRMS nor FRK have an N-terminal myristylation site.

SRC kinase adaptor phosphoprotein 2 (SKAP2) is a broadly expressed assembling platform for multiple proteins such as SRC kinases with diverse subcellular localizations (9, 10, 11). SKAP2 plays multiple functions, from control of actin-polymerization (12, 13) to podosome stabilization (14), during cellular activation by integrin clustering (15) or tyrosine kinase receptors (16). Its role in cellular differentiation and cell migration explains that this protein is associated to multiple human diseases from metastatic progression (17) to susceptibility to autoimmune diseases (18, 19, 20). SKAP2^−/−^ mouse model (21) has allowed to study the functions of this protein in different cell types such as macrophages (14, 22, 23) and neutrophils (24, 25, 26, 27). SKAP2 is composed of three domains: an N-terminal dimerization (DIM) domain, a Pleckstrin homology (PH) domain, and a SH3 domain (supplemental Fig. S1*A*). Multiple sites of tyrosine phosphorylation are found across the protein, in the linkers and their SH2 binding motifs (28, 29) as well as in the PH domain probably modulating SKAP2 capacity to bind to membranes. SKAP2 interacts directly through its SH3 domain to proline-rich motifs found in many proteins such as FYB, PRAM1, PTK2B, and FAM102A (10, 30, 31, 32). Crystallographic structure of the N-terminal region of the murine Skap2 protein shows that the DIM domain interacts with the PH domain of the same molecule, blocking access to its PIP3 (phosphatidylinositol [3–5] P3) binding pocket and dimerizes by forming a four-helix bundle (33). This dimerization was recently proposed to induce for most SRC kinases a fine-tuning between activation and an increase of their binding capacity to SKAP2 (30).

In this work, we wished to better characterize the underlying mechanism of this regulation. We show that the interaction of SKAP2 with SRC kinases depends on three modules, each of whose binding capacity is differently modulated by the activation and/or the subcellular localization of SRC kinases. Interestingly, the modular architecture of another member of the same family, SRC kinase adaptor phosphoprotein 1 (SKAP1), is different. We studied SRC kinase mutations using the SRC kinase family member HCK, which is the only one with a resolved complete 3D structure amongst the nine studied (34, 35). Our results suggest that the loss of signal for some HCK mutants with SKAP2 is due to a loss of protein stability, probably linked to proteasome-mediated degradation in which K7, play a major role. In conclusion, this work has led to a better understanding of the complex interaction between SKAP2 and SRC kinases, which may be intrinsically linked to kinase activation and trafficking. Such properties have been essential to understand the activation of other kinases with new and interesting therapeutic impact.

## Experimental Procedures

### Plasmids, Mutagenesis, BP and LR Cloning, and Luciferase Complementation Assay

Previously used plasmids and a more detailed methodology will be found elsewhere (30). The ORF of the new SKAP2 deletion mutants flanked by two gateway sites were amplified by PCR, cloned into pCR-II TOPO vector using TOPO TA cloning kit (Thermo Fisher Scientific) and transferred into pDONR207 vector. Mutagenesis of SKAP2 and HCK ORFs was performed using QuickChange Lightening Site-Directed Mutagenesis kit (Agilent Technologies) according to the manufacturer’s protocol. Supplemental Table S1 shows the new primers used for all these purposes. After each mutagenesis and PCR amplification, the insert was completely sequenced at GATC services using LightRun Barcodes (Eurofins Genomics). Sequence alignments were performed using DNA Strider (36). The ORF of SKAP2 mutants were transferred into pSPICA-N2 vector and those of SRC kinases and HCK mutants into pSPICA-N1 and pSPICA-C1 ones. The pSPICA vectors are mammalian expressing vectors designed for luciferase complementation assay. They expressed *Gaussia princeps* Luciferase fragment 1 (amino acid residues from 18 to 109) or 2 (amino acid residues from 110 to 185) at the N- or C-terminal part of the fusion protein. The luciferase complementation assay was performed as described in human HEK293T cells (37). Briefly the first day, 30,000 HEK293T cells are cultured in 100 μl of Dulbecco's modified Eagle's medium supplemented with 10% fetal bovine serum and antibiotics per well of microplate 96 wells (Greiner). One day later, cells were transfected with 100 ng GPCA plasmid pair using polyethylenimine method. The third day, culture medium was discarded, cells were washed once with 150 μl of phosphate buffer saline without calcium and magnesium (PBS) and incubated for 25 min in 40 μl of lysis buffer. Luminescence monitoring was performed after addition of native Coelenterazine on a Centro XS^3^ LB 960 microplate luminometer (Berthold Technologies). Transfections using polyethylenimine were performed at least in triplicate. Protein–protein interactions were monitored by measuring interaction-mediated normalized luminescence ratio (NLR) (37). Briefly, NLR is the ratio between the interaction signal and a control one, the sum of signals of each component of the interaction with the complementary empty hemi-luciferase expressing vector. A negative control, FAM102B, was used in each experiment as positive controls to verify the status of the SKAP2 mutants. For each experiment, a threshold was defined as NLR mean +SEM of sample FAM102B. These data are not presented in this article.

### Western Blot

Pooled triplicate samples from luciferase complementation assay diluted in Laemmli x1 buffer were passed through Qiashredder column (Qiagen) before eating at 95 °C during 5 min. Samples were loaded on a gel with PageRuler Plus Prestained Protein Ladder (ThermoFisher). After 2 h migration on a 4 to 12% NuPAGE Trisminigel (ThermoFisher Scientific) at 120 V at room temperature in x1 MES buffer, proteins were transferred to a PVDF membrane at 5 °C. After saturating the membrane with 1× PBS, Tween 0.25% 5% milk during 30 min at room temperature, an overnight hybridization at 4 °C with a primary antibody in 1× PBS, Tween 0.25%, BSA 1% buffer was performed followed by 1 h hybridization at room temperature with a secondary antibody in the same buffer. Revelation was performed on G:Box system (Syngene) after incubation with Pierce ECL2 Western Blotting substrate (ThermoFisher Scientific). Supplemental Table S2 shows the different antibodies used.

### Experimental Design and Statistical Rationale

Approximation of the mean and the variance of a ratio were performed using Taylor expansions with a null covariance between the numerator and the denominator. Two-sample z test and two-way ANOVA after log-normal transformation were used to compare these ratios. Differential interaction scatterplots and protein–protein interaction (PPI)-mutation plots were performed as described in (30). Three levels of significance were used for the *p*-value lower than 0.05 (∗), 0.01 (∗∗), and 0.001 (∗∗∗). Bonferroni correction was used for multiple testing with an initial threshold *p* = 0.05. To compare different experiments, we used modified NLR for each SRC kinases as the ratio between NLR of the SKAP2 mutant and NLR of wildtype SKAP2 protein. Each mutant experiment was repeated at least three times. Statistical analyses were performed using Stata/IC, version 10.1, (Stata Corporation). Comparison of mean to theorical value (*i.e.*, Mean ratio to 1 and its log-normal transformation to 0) and between two means was performed by using Student *t* test and unpaired Student *t* test, respectively. Comparison of mean to theorical value was also studied using binomial distribution after calculating the number of samples upper/lower to theoretical value. Comparison of two means was also performed by Kruskal–Wallis test. Parametric and nonparametric tests giving similar results, only results of the parametric are shown in Table 1. Two-way ANOVA were performed after a log-normal transformation of the modified NLR and using analytic weights. For data modeling, the full model contains nine explanatory variables and the data on the N1- and C1-fused SRC kinases were analyzed separately. For generating the variable explaining which domains are necessary to bind to either N1-fused SRC kinases or C1-fused ones, the following protocol was used. Two-way ANOVA was performed on deletion SKAP2 mutant data using five variables, each explaining the effect of a SKAP2 region. Significant variables and all their possible region combinations were used in a second two-way ANOVA. Significant variables of the second analysis were used to generate the definitive variable describing which regions are necessary for defining the binding site. An explicative model was generated from of this full model by a step-by-step procedure that removed at each step the lowest nonsignificant variable until no nonsignificant variables were present.

**Table 1:** Statistical analysis of the interaction between SKAP2 mutants and SRC kinases

### Conservation of the DIM Domain of SKAP1 and SKAP2

The N-terminal fragment of human SKAP1 and SKAP2 protein structures were predicted from I-TASSER server (38) using two crystal structures of a N-terminal fragment of murine SKAP2 containing both the helical DIM and the PH domain (ID PDB: 2OTX and 1U5E). Clustalw program (39) was used to align respectively 37 and 38 protein sequences of SKAP1 and SKAP2 from the Coelacanth to mammals. ConSurf server was used to visualize evolutionary conservation in these two macromolecules from their respective predict structure and aligned sequences using a maximum likelihood procedure. UCSF Chimera, version 1.13.1, was used to visualize the conservation of these structures using a script from ConSurf server (40, 41).

## Results

We finely analyzed the interactions between SKAP2 and nine SRC kinases. The aims were (i) to define SRC binding sites on SKAP2, (ii) to clarify how SKAP2/SRC interactions impact on the SRC activation, (iii) how subcellular localization of SRC kinases is involved in their binding to SKAP2. For that purpose, we used a luciferase complementation assay in HEK293T cells where the C-terminal hemi-gaussia2 luciferase (Gluc2) is fused at the N terminus of SKAP2 (N2-SKAP2) and the N-terminal hemi-gaussia1 luciferase (Gluc1) is fused either to the N terminus (N1) or C terminus (C1) of the SRC kinases (30). It is necessary to study the two orientations of hemi-gaussian luciferase fused to SRC kinases for evaluating the effect of their activation and/or subcellular localization on this interaction. One major assumption of this paper is that there is a general SKAP2 architecture, which defines its interaction with all the SRC kinases, and which can be studied by the mean value of their PPI signal. To assess the effect of SKAP2 deletions or mutations, the NLR (37) generated by their binding to SRC kinase has been divided by the binding of wildtype SKAP2 to the same SRC kinase. This generated a relative NLR enabling to standardize the interaction of mutated SKAP2 relative to the wildtype protein for the different SRC kinases. The interaction interface of SKAP2 with nine SRC kinases, BLK, FGR, FRK, FYN, HCK, LCK, LYN, SRMS, and YES, was studied with 21 SKAP2 mutants, eight of which are deletion mutants (Fig. 1*B*) and the others contain nonsynonymous mutations (supplemental Fig. S1*A*). Study of deletion SKAP2 mutants interacting with C1-fused SRC kinases allowed to define which SKAP2 modules are important for this interaction. We analyzed the role of both subcellular localization and activation on these interactions by comparing C1-fused SRC kinase data with those of N1-fused SRC kinases. This role was also assessed by studying the interaction of wildtype SKAP2 with 24 HCK kinase mutants from 11 mutations (supplemental Fig. S1*B*). A negative control, FAM102B, was used in each experiment as positive controls to verify the status of the SKAP2 mutants (not shown). We refined the role of the different modules by studying nonsynonymous SKAP2 mutations.

Figure 1*C* shows the initial study of the interaction between eight N2-fused SKAP2 deletion mutants (Fig. 1*B*) with N1 or C1-fused HCK SRC kinase. The signal is greater or equal for most mutants when interacting with C1-fused HCK than with N1-fused HCK but varies considerably between mutants. This suggests that the localization of the hemi-luciferase fusion disturbs these interactions without suppressing them. We will first study the eight SKAP2 deletion mutants that interact with C1-fused SRC kinases.

### The DIM Domain of SKAP2 Is Necessary and Sufficient to Bind C1-Fused SRC Kinases

**Fig. 2:**
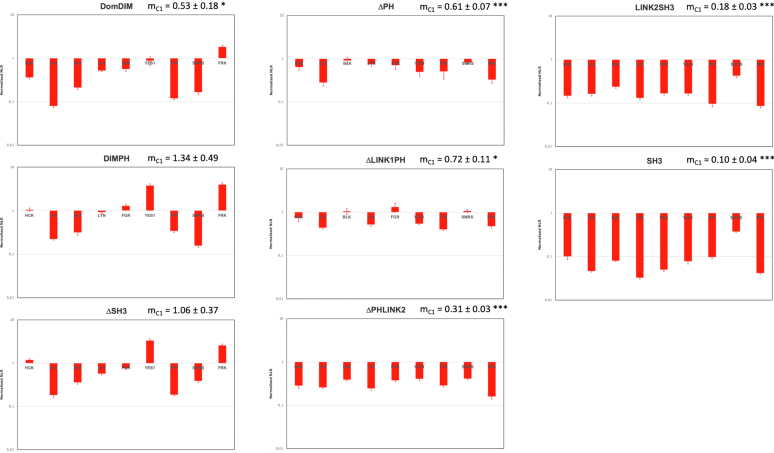
**Study of the interaction between SKAP2 deletion mutants and C1-fused SRC kinases.** Graphs show normalized NLR of the eight SKAP2 deletion mutants with C1-fused SRC kinases in a logarithmic scale. Normalized NLR is the ratio of the NLR between a SKAP2 deletion mutant and one C1-fused SRC kinase to the corresponding NLR between wildtype SKAP2 and the same C1-fused SRC kinase. For each graph, the normalized NRL mean ± SEM among SRC kinases is shown as its level of significance. DIM, domain of dimerization; NLR, normalized luminescence ratio; PH domain, Pleckstrin homology domain; SH3, Src homology 3 domain; SKAP2, SRC kinase adaptor phosphoprotein 2. *Asteriks* refer to the level of significance. ∗: *p* = 0.05; ∗∗∗: *p* = 0.001.

A previous study supports that the DIMPH SKAP2 mutant (Fig. 1*B*), which contains the DIM domain and the PH domain, is able to bind most C1-fused SRC kinases but not N1-fused ones (30). To confirm and understand the structural basis of this result, the eight SKAP2 deletion mutants (Fig. 1*B*) were studied with the nine C1-fused SRC kinases (Fig. 2). A mutation is considered to destroy an interaction if the average of the relative NLRs among the nine SRC kinases is below 0.25. Only the two SKAP2 mutants lacking the DIM domain (Link2SH3 and SH3), did not bind to the C1-fused SRC kinases, supporting a necessary role of this domain. A similar binding pattern is observed in three SKAP2 mutants, DomDIM, DIMPH and ΔSH3, with the highest signals for YES and FRK, middle signals for HCK, LYN, and FGR, and the lowest signal for BLK, LCK, FYN, and SRMS. These data support that the DIM domain is sufficient for the interaction but that other regions such as the SH3 domain also play a role. The study of the interaction between N2-SKAP2 and N1-fused SRC kinases has further defined these SKAP2 modules.

### SKAP2 Modular Organization for the Binding to N1-Fused SRC Kinases Depends on Three Modules

**Fig. 3:**
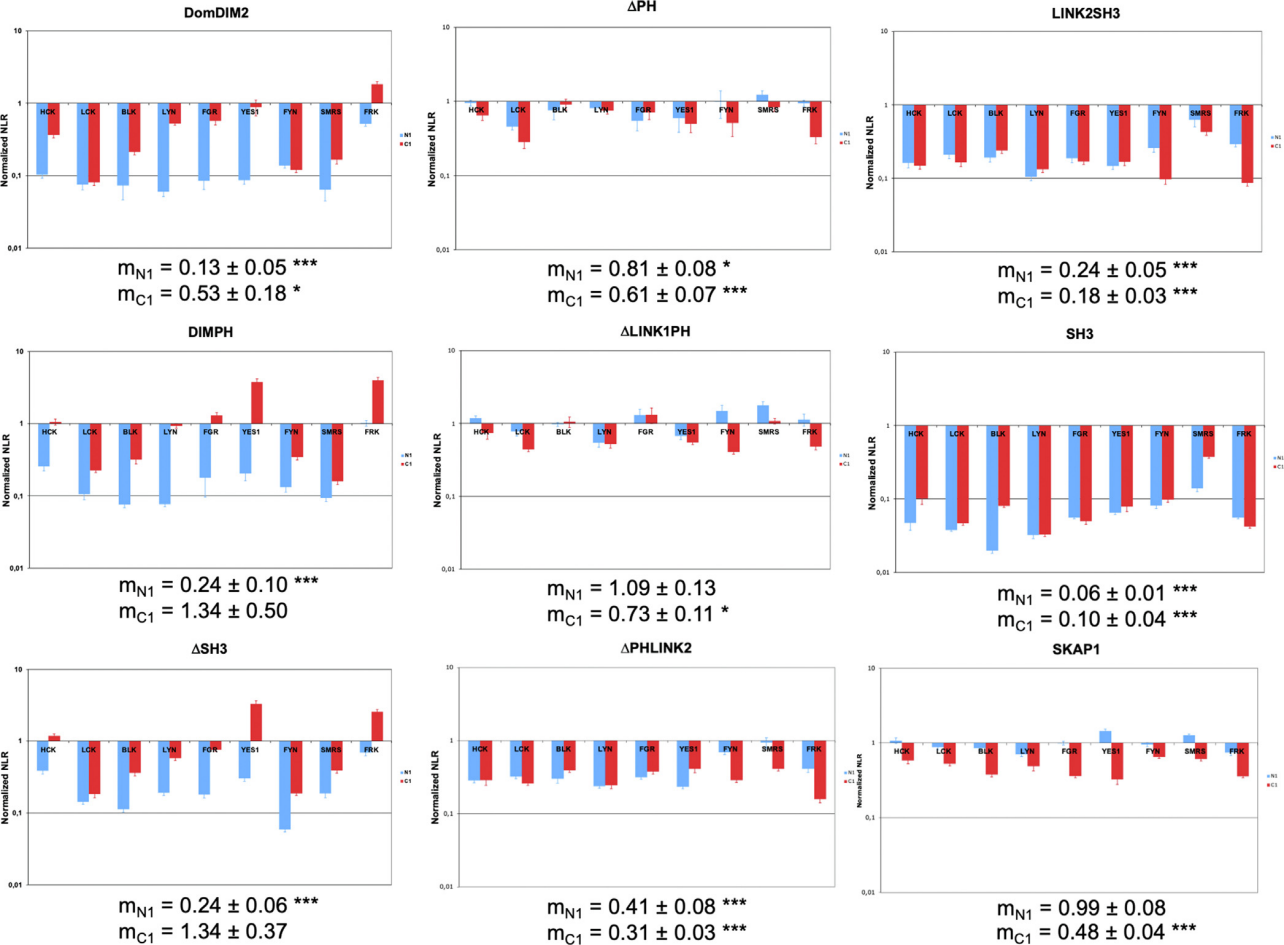
**Study of the interaction between SKAP2 deletion mutants and either N1-fused or C1-fused SRC kinases.** Graphs show normalized NLR of the eight SKAP2 deletion mutants with N1-fused (*blue*) and C1-fused (*red*) SRC kinases in a logarithmic scale. Normalized NLR is the ratio of the NLR between a SKAP2 deletion mutant and one SRC kinase either N1-fused or C1-fused to the corresponding NLR between wildtype SKAP2 and the same SRC kinase fused at the same position. For each graph, the normalized NRL mean ± SEM among SRC kinases is shown as its level of significance. DIM, domain of dimerization; NLR, normalized luminescence ratio; PH domain, Pleckstrin homology domain; SH3, Src homology 3 domain; SKAP2, SRC kinase adaptor phosphoprotein 2. *Asteriks* refer to the level of significance. ∗: *p* = 0.05; ∗∗∗: *p* = 0.001.

When the Gluc1 fragment is fused to the N terminus of SRC kinases, five SKAP2 deletion mutants, DomDIM2, DIMPH, ΔSH3, SH3, and LINK2SH3, completely lost their binding capacity for SRC kinases and only ΔLINK1PH mutant showed no decrease in signaling compared to wildtype SKAP2 (Fig. 3). Data on ΔLINK1PH mutant support an important role for other SKAP2 domains, *i.e.*, the DIM and SH3 domains and the linker 2. As previous results have shown that the DIM domain alone did not bind to N1-fused SRC kinases, these results suggest that the three modules DIM, SH3, and linker2 are necessary. Note that the two non-myristate SRC kinases, SRMS and FRK, do not have any particular profile compared to myristate ones, which supports that the localization defect induced by the N1 fusion is not prohibitive for the study of these interactions. Furthermore, differential interaction scatterplots, comparing the mean value of luminescence (NLR) induced by the interaction of each SKAP2 mutant to that induced by the interaction of wildtype SKAP2, confirmed the major effect of the Gluc1 orientation on DomDIM2, DIMPH, and ΔSH3 SKAP2 mutants (supplemental Fig. S3). These results support that the SKAP2 modular organization for binding to SRC kinases has its properties depending on the localization of the hemi-luciferase fusion.

### Role of SKAP2 Dimerization

**Fig. 4:**
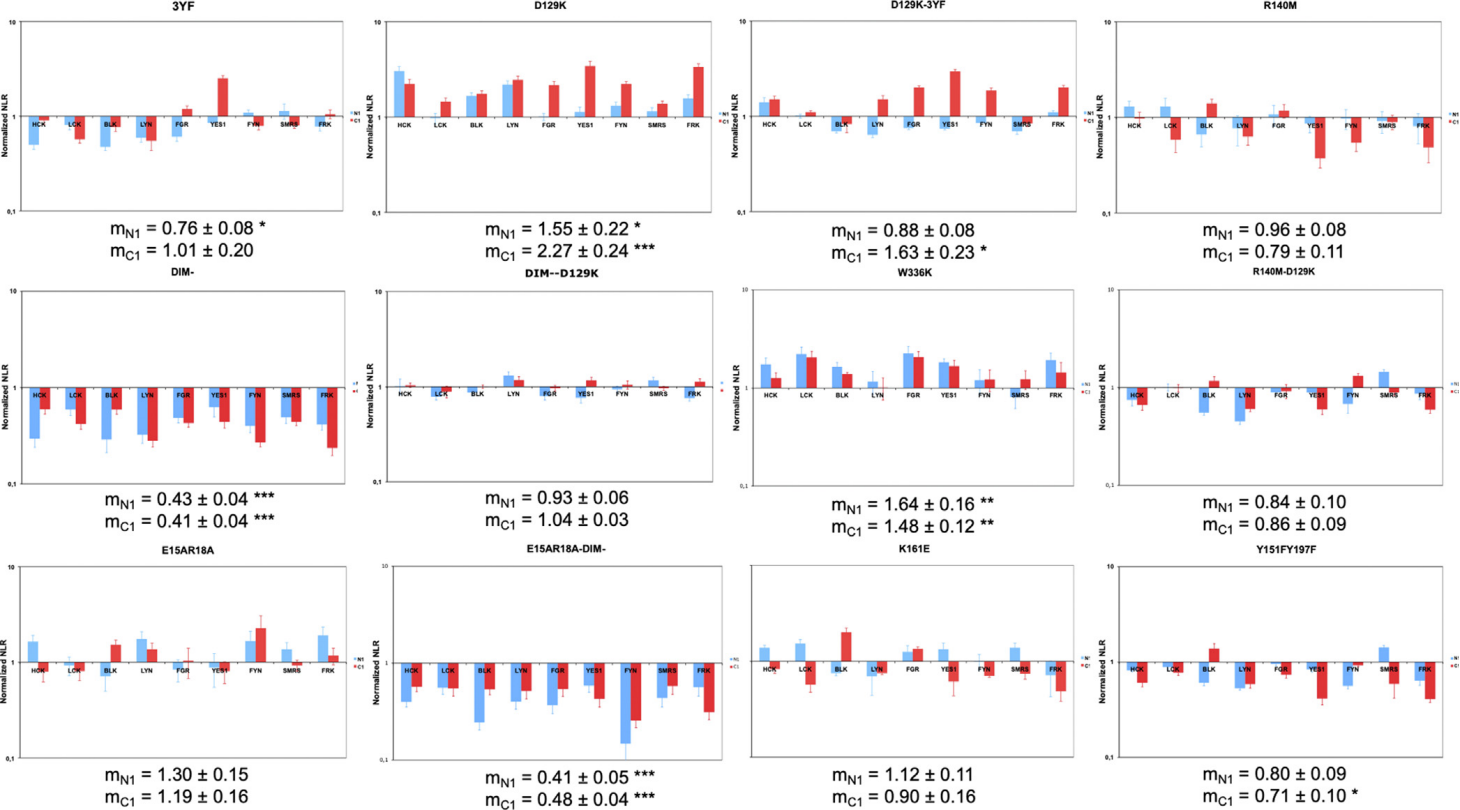
**Effect of SKAP2 point mutations on interaction with SRC kinases.** Each graph shows normalized NLR of each of the 12 SKAP2 mutants with N1-fused (*blue*) and C1-fused (*red*) SRC kinases in a logarithmic scale. Normalized NLR is the ratio of the NLR between a SKAP2 deletion mutant and one SRC kinase either N1-fused or C1-fused to the corresponding NLR between wildtype SKAP2 and the same SRC kinase fused at the same position. For each graph, the normalized NRL mean ± SEM among SRC kinases is shown as its level of significance. DIM, domain of dimerization; NLR, normalized luminescence ratio; SKAP2, SRC kinase adaptor phosphoprotein 2. *Asteriks* refer to the level of significance. ∗: *p* = 0.05; ∗∗: *p* = 0.01; ∗∗∗: *p* = 0.001.

To exclude an effect of dimerization specifically on the binding of activated C1-fused SRC kinases to DIM domain, a dimerization-invalidated mutant of SKAP2, DIM^−^, bearing the V24D, F27G, and V28E mutations (supplemental Fig. S1*A*) (33) was studied (Fig. 4 and supplemental Fig. S4). A similar significant decrease of interaction signal of both N1-fused and C1-fused SRC kinases to SKAP2 DIM^−^ mutant was detected without suppressing their binding capacity. This result strongly suggests that dimerization influences binding but in a different way to that of the DomDIM2 SKAP2 mutant, which has lost its ability to bind only to N1-fused SRC kinases.

### Limited Role of Conserved Tyrosines

Classical views suggest that the binding of SRC kinases to SKAP2 occurs primarily through the SH2 domain of SRC kinases and one of the two phosphorylated tyrosine Y237 or Y261 motifs of SKAP2 (9, 11, 16). Our results of the SKAP2 deletion mutants do not support this hypothesis. To directly test it, a SKAP2 3YF mutant bearing three nonsynonymous mutations, Y75F, Y237F, and Y261F, was studied (supplemental Fig. S1*A*). Only a slight decrease of the signal for N1-fused SRC kinases was detected (Fig. 4 and supplemental Fig. S4). This result confirms a limited role of these three tyrosines.

### Inactivation of the Classical SH3 Binding Site has a Completely Different Effect Than the SH3 Deletion

The SH3 domain of SKAP2 interacts with proline-rich helix of its partners for which its tryptophan 336 is required (10). Interestingly, the study of W336K SKAP2 mutant (supplemental Fig. S1*A*) shows a significant increase of signal for both N1-fused and C1-fused SRC kinases (Fig. 4). The SH3 deletion has an opposite effect, significantly decreasing the signal for N1-fused SRC kinases (Fig. 3) and inducing a specific pattern for C1-fused ones (Fig. 2).

### Role of the PH Domain by Studying Nonsynonymous Mutations

The PH domain of SKAP2 is known to bind to the phosphatidyl inositol 3,4,5 triphosphate (PI[3,4,5]P_3_). It also interacts with the DIM domain of the same molecule, which prevents the binding to PI[3,4,5]P_3_ (33). Three mutations in the PH domain known to affect the interaction with the DIM domain, D129K (12, 13, 33), R140M (33), and K161E (42) and four new mutations located in the DIM domain (E15A, R18A), or in the PH domain (Y151F and Y197F) (supplemental Fig. S1*A*) were studied alone or in combination. The R140M and K161E mutations are known to affect the binding of PI[3,4,5]P_3_ and D129K to prevent the intramolecular interaction with DIM domain. Crystallographic data on the murine Skap2 support that E15A and R18A mutations located in the DIM domain prevent its interaction with the PH domain. Literature curation supports the view that Y151F and Y197F mutations affect the binding to PI[3,4,5]P_3_ and possibly to the DIM domain (33). As shown in Figure 4 and supplemental Fig. S4, these mutations have a limited effect on the interaction with SRC kinases, except D129K, which surprisingly increased signaling primarily with C1-fused SRC kinases. No of them has lost their binding capacity for SRC kinases and only some of them have a significant decrease.

In conclusion, the data presented in the last four paragraphs show that nonsynonymous mutations alone or in combination have a weaker effect on SRC kinase binding than deletion of the corresponding binding domain or region, consistent with a modular organization. It is noteworthy that also in this case, the two nonmyristate SRC kinases, SRMS and FRK, do not have a particular profile compared to the myristate kinases, confirming that the data with the N1 fusion provide useful information for studying the molecular architecture of SKAP2.

### Role of SRC Kinase Activation on the Interaction with SKAP2

Furthermore, the study of the SKAP2 paralog, SKAP1, supports an even more complex binding of SRC kinases (Fig. 3). In contrast to previous results with deletion SKAP2 mutants, the PPI signal of the N1-fused SRC kinases with SKAP1 was higher than that of the C1-fused SRC kinases, while similar SRC kinases binding was expected from the high homology between SKAP1 and SKAP2. To reconcile these data, we took account that SRC kinases adopt different conformations affecting their kinase activity (4, 43) and that PPI signal between SKAP2 and SRC kinases was higher for activated than inactivated HCK kinase in our initial work (30). We now suppose (i) that the orientation of the fused hemi-luciferase differently affects the conformations and activation of SRC kinases, (ii) that SKAP2 binding sites for SRC kinases exhibit different binding properties depending on their conformations, and (iii) the latter is not the case for SKAP1. These hypotheses have three consequences: (1) the DIM domain of SKAP1 binds less efficiently C1-fused SRC kinases than that of SKAP2; (2) the DIM domain of SKAP2 has a greater increase of its binding capacity for activated HCK mutants than the wildtype SKAP2 and the DIM domain of SKAP1, independently of the orientation for the hemi-luciferase; (3) different properties of the SKAP2 binding site can be defined by studying N1-fused and C1-fused SRC kinases with SKAP2 deletion mutants. Results of the two first paragraphs are already consistent with the last hypothesis.

### Functional Differences Between the DIM Domain of SKAP1 and SKAP2

**Fig. 5:**
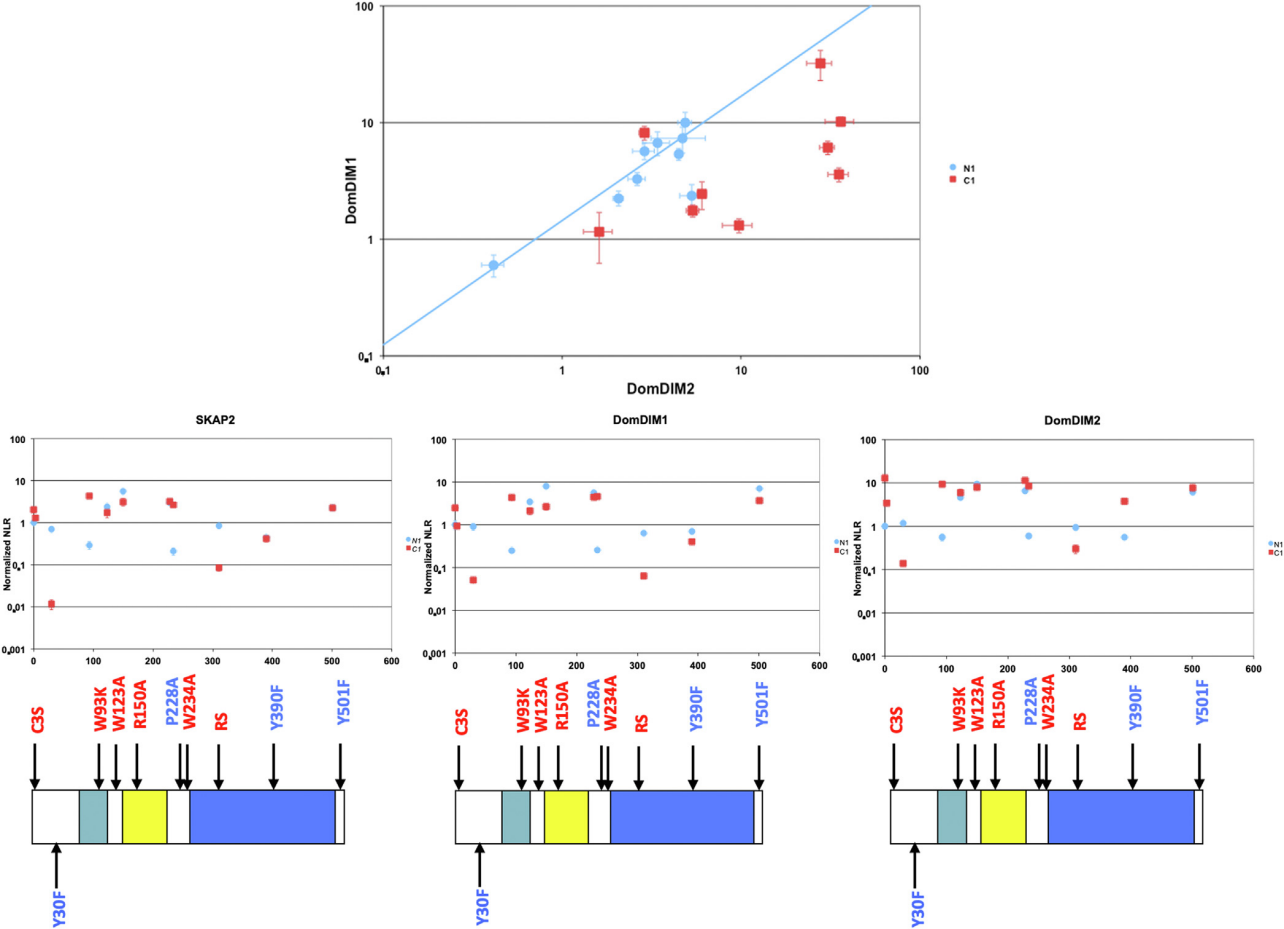
**Comparison of binding properties from DIM domains of SKAP1 and SKAP2 proteins.***Upper panel*, differential interaction scatterplot of DIM domain of SKAP1 protein (DomDIM1; y-axis) *versus* DIM domain of SKAP2 protein (DomDIM2; x-axis). Interactions not affected by the mutation are aligned on the diagonal. For DomDIM1, PPI disruptive mutations are located on the *lower right-hand quadrant* in contrast to mutations stabilizing interaction that are located on the *upper left-hand one*. Interactions for N1-fused SRC kinase are in *blue circle* and those for C1-fused SRC kinase are in *red square*. Error bar: standard error to the mean (SEM). A robust linear regression, which takes account of outliers, was performed on data using mmregress Stata module. Linear regression equation is log10(DomDIM1) = 1.065∗ log10(DomDIM2) ± 0.162 for N1-fused SRC kinases. *Lower panels*, PPI-mutation plot of respectively, SKAP2 protein, DomDIM1, and DomDIM2 domains with N1-fused (*blue*) and C1-fused (*red*) HCK mutants and the corresponding schematics of HCK organization. In PPI-mutation plot, Y-axis represents NLR of each HCK mutant in a logarithmic scale and X-axis, the position of the mutation. NLR are normalized according to that of HCK-N1. *Arrows* showed the position of the HCK mutations; wildtype and replacement amino acid identities at mutated position involved in domain or motif inactivation are indicated in *red* and *blue* respectively. DIM, domain of dimerization; HCK, hematopoietic cell kinase; NLR, normalized luminescence ratio; SKAP1, SRC kinase adaptor phosphoprotein 1; SKAP2, SRC kinase adaptor phosphoprotein 2.

A phylogeny analysis of each SKAP1 and SKAP2 protein allows to define the amino acid conservation in both structures using CONSURF website (40). A stronger conservation of the DIM domain of SKAP2 than that of SKAP1 was detected (supplemental Fig. S2). This loss of SKAP1 conservation during evolution suggests that the binding capacity to SRC kinases by its DIM domain has changed over time compared to the DIM domain of SKAP2. This gives a molecular support that the DIM domain of SKAP1 has a lower binding capacity for C1-fused SRC kinases than that of SKAP2. A differential interaction scatterplot of data from DomDIM1 SKAP1 mutant (y-axis) *versus* those from DomDIM2 SKAP2 mutant (x-axis) supports also the first consequence of the hypothesis (Fig. 5). Each point is the data of a N1-fused (blue) or C1-fused (red) SRC kinase interacting with either the DIM domain of SKAP2 or that of SKAP1. The data of N1-fused SRC kinases were aligned along the diagonal supporting that their capacity of binding is similar for both SKAP1 and SKAP2 proteins. In contrast, most data of C1-fused SRC kinases are in the lower right-hand quadrant in agreement with a higher binding capacity for DomDIM2 SKAP2 mutant than for DomDIM1 SKAP1 mutant.

Study of HCK mutants agrees with the second consequence (Fig. 5). First, note that HCK mutations known to mimic the activated kinase such as W93K, R150A, P228A, and Y501F increase the binding signal to wildtype SKAP2, DomDIM1, or DomDIM2 independently of the N1 or C1 fusion. The only exception is N1-W93K for which an explanation will be given later. This supports that both configurations of the hemi-luciferase fusion in SRC kinases allow to study the molecular architecture of SKAP2 independently of their effects on kinases, since both detected an increase of binding capacity for activated HCK mutants. Second, the PPI signal strength of the HCK mutants known to mimic the activated kinase and C1-fused wildtype HCK was stronger for the DIM domain of SKAP2 than for either the DIM domain of SKAP1 or wildtype SKAP2 itself. These results support that the DIM domain of SKAP2 has a greater increase of its binding capacity for activated HCK mutants and C1-fused wildtype HCK than the DIM domain of SKAP1 and SKAP2 itself. These results are consistent with the first two consequences described above.

### Statistical Validations

Previous hypotheses were confirmed using univariate analyses combining at least three experiments. For each SKAP2 mutant, three effects were tested on N1-fused SRC kinases and on C1-fused ones comparing to theorical mean and on the difference between the signal of the C1-fused SRC kinases and that of N1-fused ones. Results were considered statistically significant only after Bonferroni’s correction (Table 1).

Mutants with a loss of interaction were the same as in the previous analysis except for N1-fused LINK2SH3, which was just above the threshold (0.272 *versus* 0.25). Comparison of the deletion mutants shows that (i) for ΔSH3, DIMPH, the signal significantly decreases only for N1-fused SRC kinases and (ii) for only one mutant, ΔLINK1PH SKAP2, the signal significantly decreases specifically for C1-fused SRC kinases. For all other deletion mutants, the signal significantly decreases for both N1-fused and C1-fused SRC kinases. For DomDIM and ΔSH3 mutants, the signal for C1-fused SRC kinases was significantly higher than those for N1-fused ones. These results strongly support that the DIM domain is sufficient to interact with C1-fused SRC kinases even if other domains play a role. For example, the SH3 domain explains the similar binding pattern of C1-fused SRC kinases among the three SKAP2 mutants, DomDIM, DIMPH, and ΔSH3. For the binding of N1-fused SRC kinases, both DIM and SH3 domains are necessary. Finally, the second linker between the PH and the SH3 domains probably affects SRC kinase binding independently of the fusion position since the signal decreases more for the ΔPHLINK2 mutant than for the ΔPH and ΔLINK1PH mutants. The fact that the signal of SKAP1 decreases specifically for C1-fused SRC kinases agrees with previous hypotheses. Other statistical analyses for single point mutants show that for W336K and D129K SKAP2 mutants, the signal significantly increases while for DIM^−^ SKAP2 mutant, it significantly decreases independently of the orientation of the fused Gluc1. For 3YF SKAP2 mutant, only the signal of N1-fused SRC kinases decreases. For D129K-3YF mutant, the signal significantly increases only for C1-fused SRC kinases. In contrast, it significantly decreases for DIM^—^D129K mutant. In conclusion, these results give a statistical support to our previous analyses.

Second, a statistical modeling was performed taking account both literature curation and our results. Nine variables were generated, describing function possibly affected by mutations and a status for each variable was assigned for each mutant. The two variables describing how SKAP2 protein interacts with N1-fused or C1-fused SRC kinases were generated according to the following protocol: first, which of the five domains or regions significantly decreases the signal and second, which associations between the regions highlighted in the first part of this analysis significantly decrease the signal. The study of C1-fused SRC kinases data shows that the binding capacity of SKAP2 depends mainly on the presence of the DIM domain but is modulated by the presence of the SH3 domain and of the Link2 interdomain. The analysis of the N1-fused SRC kinases data shows that the binding site of SKAP2 protein depends on the presence of three regions, DIM, Link2, and SH3 altogether. A two-way ANOVA analysis was carried out after lognormal transformation of the dependent variable on these nine variables with either N1-fused or C1-fused SRC kinase data. This transformation was chosen since data are linearized after log-log transformation in differential interaction scatterplots (see supplemental Figs. S3 and S4). An explicative model was generated from a full model by a step-by-step procedure that removed at each step the lowest nonsignificant variable until no nonsignificant variables were present (Table 2).

**Table 2:** Statistical models of the interaction between SKAP2 mutants and SRC kinases

Variable	N1-Loc					C1-Loc				
	Partial SS^b^	df	MS	F (sense)	*p*^a^	Partial SS^b^	df	MS	F (sense)	*p*^a^
Explanatory model	595.44	5	119.09	255.6	1.09 10^−142^	353.62	8	44.20	112.62	3.40 10^−112^
Loss of link between DIM-PH					NS	5.00	1	5.00	12.73 (+)	0.0004
Gain of PH binding site	3.13	1	3.13	6.71 (+)	0.0098	5.13	1	5.13	13.07 (+)	0.0003
SKAP1					NS	5.92	1	5.92	15.09 (−)	0.0001
Loss of PH binding site					NS	4.59	1	4.59	11.70 (−)	0.0007
DIM-	10.17	1	10.17	21.82 (−)	3.74 10^−6^	15.37	1	15.37	39.16 (−)	3.85 10^−10^
W336K	7.79	1	7.79	16.72 (+)	4.96 10^−5^	11.37	1	11.37	28.96 (+)	5.41 10^−8^
3YF	5.33	1	5.33	11.44 (−)	0.0008					NS
SKAP2 module 2	431.44	1	431.44	926.01 (−)	1.57 10^−121^	ND	ND	ND	ND	ND
SKAP2 module 1	ND	ND	ND	ND	ND	147.17	2	73.58	187.48 (−)	1.59 10^−63^
Residual	264.97	570	0.47			222.54	567	0.39		

a NS: nonsignificant (deleted variable during the procedure of minimization).

b ND: not done.

Only three variables have similar effect on both N1-fused and C1-fused SRC kinase, the gain of function associated to D129K and W336K mutations, which increase the signal of interaction, the DIM^−^ mutation, which has an opposite effect. The 3YF mutation, Y75F, Y237F, and Y261F, specifically, decreases the signal of interaction with N1-fused SRC kinases. Mutation located in the PI[3,4,5]P_3_-binding pocket of the PH domain and SKAP1 protein specifically decrease the signal of interaction with C1-fused SRC kinases and the loss of association between the DIM and the PH domain, which has an opposite effect. In conclusion, this analysis strongly supports a modular organization of SKAP2 for binding to SRC kinases being composed of three modules, the DIM and SH3 domains, and the interdomain between the PH and the SH3 domains, which each module having its SRC kinase binding capacity modulated differently by their activation and/or their subcellular localization. Also, note that the binding capacity of SKAP1 to SRC kinases decreases with C1-fused SRC kinases, in agreement with the loss of binding of its DIM domain (Fig. 3).

### Analysis of HCK Mutants

Two nonexclusive hypotheses from literature curation might explain differences between N-terminal– and C-terminal–fused SRC kinases: a different subcellular localization and their level of activation. First, most SRC kinases such as HCK, are located to membranes by N-terminal myristylation and/or palmitoylation (7, 8). This process should be blocked by the N-terminal fusion of a hemi-luciferase. Second, a major process of activation is the dephosphorylation of Tyr501, which suppresses its intramolecular binding to the SH2 domain (3, 4). C-terminal fusion of a hemi-luciferase might decrease the phosphorylation of Tyr501 by CSK kinase by masking a C-terminal binding site. To dissociate these two nonexclusive effects, we studied different HCK mutants. In our previous paper (30), we have shown that the binding capacity of SKAP2 for SRC kinases specifically increases with their activation. We repeat this experiment by studying HCK mutants mimicking an activated kinase state (W93F, R150M, P228A, Y501F mutations alone or in combination). Two HCK mutations affecting its subcellular localization have also been studied only with C-terminal fusion of Gluc1: the G2A mutation suppressing any possibility of lipidation and the C3S mutation affecting palmitoylation. Interestingly, the kinase domain spontaneously adopts an active conformation in presence of its N-terminal region containing tryptophan 234, which induces rearrangement of two hydrophobic structures, the catalytic and the regulatory (RS) spines (35, 44, 45, 46, 47, 48). Twenty HCK mutants generated from 11 mutations (supplemental Fig. S1*B*) and fused with hemi-luciferase1 at their N or C terminus were tested for their binding to SKAP2 protein (supplemental Fig. S5). HCK mutants bearing known mutations that activate kinase activity have a higher signal of interaction with SKAP2 than wildtype HCK independently of the Gluc1 orientation. N1-fused W93F HCK mutant is the only discordant result. These results confirmed that both fusion localizations detected the increase of PPI signal induced by SRC activation supporting their interest to define SKAP2 modular architecture.

**Fig. 6:**
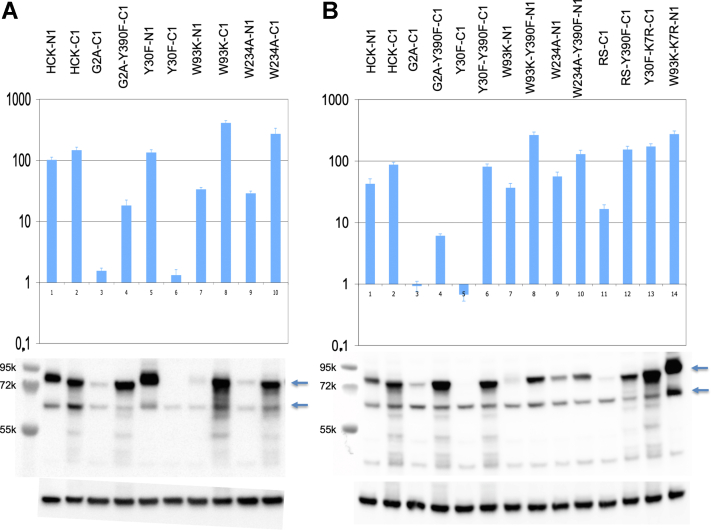
**Role of protein stability on the signal of interaction between five HCK mutants and SKAP2.***A*, differences related to hemi-luciferase localization in HCK mutants. *B*, effects of Y390F and K7R mutations on these differences. In *A* and *B*, the *upper panel* shows normalized NLR of the samples loaded on the polyacrylamide gel on the same order. The *middle panel* shows the Western blot with a polyclonal antibody against Gaussia princeps luciferase detecting both SKAP2-N2 and HCK-N1 or HCK-C1 WT and mutant proteins. *Left lane*, PageRuler Plus Prestained Protein Ladder with their corresponding molecular weight. *Upper arrow*, HCK-N1 and HCK-C1 WT and mutant proteins. *Lower arrow*, SKAP2-N2. The *lower panel* shows the Western blot on the same membrane using an antibody against GAPDH. DIM, domain of dimerization; HCK, hematopoietic cell kinase; NLR, normalized luminescence ratio; SKAP2, SRC kinase adaptor phosphoprotein 2.

Four mutations, Y30F, W93F, W234A, and RS, induced differential signals of HCK kinase depending on the orientation of the fused luciferase fragment. RS mutation is a couple of mutations, I309A-T311A, located in the regulatory spine and known to inactivate the kinase activity (49, 50). Also, the signal completely disappeared for C1-fused G2A HCK mutant. However, these effects are not mainly due to a difference of binding capacity but to a difference of protein steady state level (Fig. 6). N2-SKAP2 and N1- or C1-HCK mutants and wildtype were detected by a polyclonal antibody against *Gaussia princeps* luciferase. The level of HCK protein is followed by the decrease of the upper band (HCK) relative to the lower band (SKAP2) as well as by the appearance of small-sized degradation products. The level of HCK protein and the binding capacity were recovered by adding a second Y390F mutation to the four mutants of HCK C1-Y30F, N1-W93K, N1-W234A, C1-RS, and only protein level was rescued for C1-G2A (Fig. 6, *A* and *B*, and supplemental Fig. S5). Literature curation (6, 29) as well as the use of an expression vector support than the main effect on HCK steady state is due to degradation. Interestingly, small-sized degradation products are detected with C1-fused wildtype and mutant HCK but not with N1-fused wildtype and mutant HCK suggesting an N-terminal degradation. To support a role of ubiquitination, we studied K7R mutation in which degradation possibly does not occur. For all the mutants tested, C1-fused Y30F HCK, N1-fused W93K HCK, and N1-fused W234A HCK, a full binding capacity was recovered when K7R mutation was present as well as protein stability for two of them (Fig. 6*B* and supplemental Fig. S7). In conclusion, our present work did not confirm a role of W93K HCK mutation on SKAP2 binding as suggested in our original work but suggested that different HCK mutations have an effect on protein stability. The following model supported by literature suggests that ubiquitination of lysine 7 after the binding of Cbl ubiquitin ligase by its SH2 domain on phosphorylated tyrosine 390 motif activates proteasome degradation (6, 29). Interestingly, the binding capacity to SKAP2 is poorly recovered for G2A HCK mutant suggesting that membranous localization of SRC kinases is necessary to bind SKAP2 at least for this HCK isoform. Note also that the steady state of N2 SKAP2 is slightly affected depending on HCK mutants.

## Discussion

In a previous paper (30), PPIs between SKAP2 and most of its partners were finely analyzed. However, for SRC kinases, we could show only a limited effect of the SH2 domain of SRC kinases, at least for HCK and those of three phosphorylated tyrosines of SKAP2, Y75, Y237, and Y261. Interestingly, DIMPH, a deletion mutant of SKAP2, has completely lost its capacity to bind N1-fused SRC kinases but has retained it for most C1-fused SRC kinases. The purpose of the present paper is to solve how SKAP2 and SRC kinases interact. One major difficulty to study SRC kinases comes from their activation, which seems to increase the binding to SKAP2 for the most part independently of the classical binding sites inside their SH2 and SH3 domains (supplemental Fig. S5). In particular, the PPI signal of SKAP2 for wildtype HCK is lower than that for W93K R150M HCK double mutant, in which each classical binding site for both the SH3 and SH2 domain were inactivated. So, we decided to concentrate our efforts on SKAP2 binding sites. The interactions of 21 SKAP2 mutants, eight of which are deletion mutants with nine SRC kinases fused with hemi-Gaussia luciferase at either their N or C terminus were studied. The localization of the fused hemi-luciferase on SRC kinases probably affects both their activation and subcellular localization. This was the main reason to study N1-fused SRC kinases: evaluating the effect of both activation and subcellular localization by comparing data obtained with N1-fused SRC kinases and those with C1-fused ones. Note also that the analysis of the data defining SKAP2 architecture was simpler for the N1-fused SRC kinases than the C1-fused ones. Furthermore, rare N-terminal fusions of FGR kinases have been reported during cancers (51) supporting to directly evaluate the effects of these fusions by our approach. Lastly, we would like to emphasis that cytosolic SRC kinase family member localization has been evocated in many reviews (52) and has been confirmed experimentally (53, 54, 55, 56). Further studies of HCK mutants and of SRMS and FRK, two non-myristyl SRC kinases confirmed the role of their activation and subcellular localization and supported that their effects on SKAP2 modular architecture might be deduced from the comparison of results on both localization of the protein fusion. Direct NLR between wildtype SKAP2 and the nine N1 or C1-fused wildtype SRC kinases gives a last insight to support this view (supplemental Fig. S6). This signal greatly varies depending on the SRC kinases and the localization of the hemi-luciferase fusion. Interestingly, the fusion localization has a small effect for the two non-myristate SRC kinases, SRMS and FRK, but also for four other myristate SRC kinases, FYN, LCK, LYN, and YES1. In contrast, the signal was higher for C1-fused than N1-fused proteins for three SRC kinases, BLK, FGR et HCK. This pattern differing from the previous one defined from the study of the three deletion mutants, DomDIM, DIMPH, and ΔSH3, supports that luciferase complementation assay associated to mutagenesis is an efficient tool to study this interaction even if each fusion localization has its own limitation. The different patterns support the view that some important differences exist among SRC kinases even if a global SKAP2 modular architecture emerges from the present work. Study of SKAP2 deletion mutants shows that the DIM domain is necessary and sufficient to bind most C1-fused SRC kinases. However, this result does not explain how SKAP1 protein, a paralog of SKAP2, binds SRC kinases. We were thus supposing that the SKAP2 modular organization for binding to SRC kinases depends on their conformations and localizations, in contrast to SKAP1 one. This main difference is due to different properties of their DIM domains. Three different results support this hypothesis: First, the strength of interaction of most SRC kinases for the DIM domain of SKAP2 is higher than for the DIM domain of SKAP1; second, the strength of interaction of activated HCK mutants is higher for the DIM domain of SKAP2 than for the DIM domain of SKAP1 and wildtype SKAP2 independently of the fusion localization; third, this SKAP2 modular organization containing the DIM and the SH3 domain, and the second linker is modulated depending on the activation status and the subcellular organization of SRC kinases. In addition to previous discussion, these three independent confirmations support that this protein–protein interaction study is insight full despite the positional effect of the fusion protein on SRC kinases.

Our present results did not support a role for the SH3 domain of HCK in initiating the interaction with SKAP2 protein. In our initial study (30), the protein level of HCK mutants was not evaluated. The present data with literature curation support that at least five mutations affect HCK protein stability depending on the orientation of hemi gaussia1 fusion (Fig. 6 and supplemental Fig. S7). Previous articles have reported that SRC kinase activation induces proteasome degradation after the binding of Cbl ubiquitin ligase by its SH2 domain on phosphorylated tyrosine 390 binding motif (6). Our data agree with this model and suggest a degradation where ubiquitination of lysine7 plays a central role.

A statistical modeling supports that the SKAP2 modular architecture for SRC binding is composed of three modules, the DIM and the SH3 domain, and the interdomain between the PH and SH3 domains with some modulations depending on different factors. Our results confirmed and extended the limited role of the three SKAP2 tyrosines, Y75, Y237, and Y261 (Fig. 4 and supplemental Fig. S4). However, we are not able to conclude if this limited effect affects directly or indirectly the binding to SRC kinases. Interestingly inactivation of SH3 binding capacity by W336K mutation, which increases the signal of interaction with SRC kinases, independently of the orientation of hemi gaussia1 fusion, has a completely different effect than the SH3 deletion (Table 1). This suggests a new hypothesis where SKAP2 would be maintained away from SRC kinases through interaction of its SH3 domain with other interactors, independently of its role for their SRC binding. The study of mutations inside the PI[3,4,5]P_3_-binding pocket of the PH domain strongly suggests that mutations of this site modulate SKAP2 interaction with SRC kinases by decreasing its localization close to SRC kinases (Table 2). Interestingly, D129K SKAP2 mutation possibly increases the strength of the interaction with SRC kinases by two different mechanisms: it makes the PI[3,4,5]P_3_-binding pocket accessible to PI[3,4,5]P_3_ and/or induces another function, possibly a new site for positively charge lipids (33, 57, 58). Our statistical modeling suggests that both mechanisms affect this binding (Table 2). Study of the DIM^−^ SKAP2 mutant shows the positive role of dimerization in the PPI strength of both N1-fused and C1-fused SRC kinases even if the mechanism is less clear. One interesting hypothesis is a positive allosteric interaction modulating the SKAP2 modular architecture.

A similar structure to the SKAP2 dimer consisting in a four-helixes bundle has been already reported to have a central role in kinase activation through allosteric control (59). Future work will determine whether this is a new example of a protein–protein interaction that allosterically modulates kinase activation and/or trafficking with interesting new therapeutic consequences such as in oncology (60, 61, 62).

## Conclusions

In this article, we show that the modular architecture of SKAP2 for binding to SRC kinase family involves its DIM domain, the SH3 domain, and the second interdomain. The binding capacity of each module to SRC kinases differs depending on the localization and the activation of the kinases. Also, we propose that the stability of SRC kinase family member HCK is affected by proteasome-mediated degradation. These data are necessary to understand how SKAP2, which is associated multiple diseases from susceptibility to autoimmune disorders to some cancer prognostics, affects SRC kinase functions and what is its relationship with its own functions. Modulating specifically this interaction might have interesting therapeutic consequences.

## Data Availability

The datasets used and/or analyzed during the current study as well as mutants generated in the present study are available from the corresponding author on reasonable request.

## Ethics Approval and Consent to Participate

This research has received an OGM agreement from the Ministère de l’Education Nationale (Dossier DUO n°9216).

## Supplemental data

This article contains supplemental data.

## Conflict of interest

The authors declare no competing interests.
