# Supplementary material for: SKAP2 Modular Organization Differently Recognizes SRC Kinases Depending on Their Activation Status and Localization
Source: Mol Cell Proteomics. 2022 Nov 21;22(1):100451. doi: 10.1016/j.mcpro.2022.100451 (PMC9792355; doi:10.1016/j.mcpro.2022.100451)

Figure S1: Structural domain organization of SKAP2 and HCK proteins.

For each protein, the delineation of domain boundaries is indicated. Wild type and replacement amino acid identities at mutated position involved in domain or motif inactivation are indicated in red and blue respectively. (A) Schematic of SKAP2 organization with its three domains: dimerization domain (DIM), Pleckstrin homology domain (PH), and SRC homology 3 domain (SH3). (B) Schematic of HCK protein organization with its three domains: SRC homology 3 domain (SH3), SRC homology 2 domain (SH2) and tyrosine kinase catalytic domain (TYRKC).

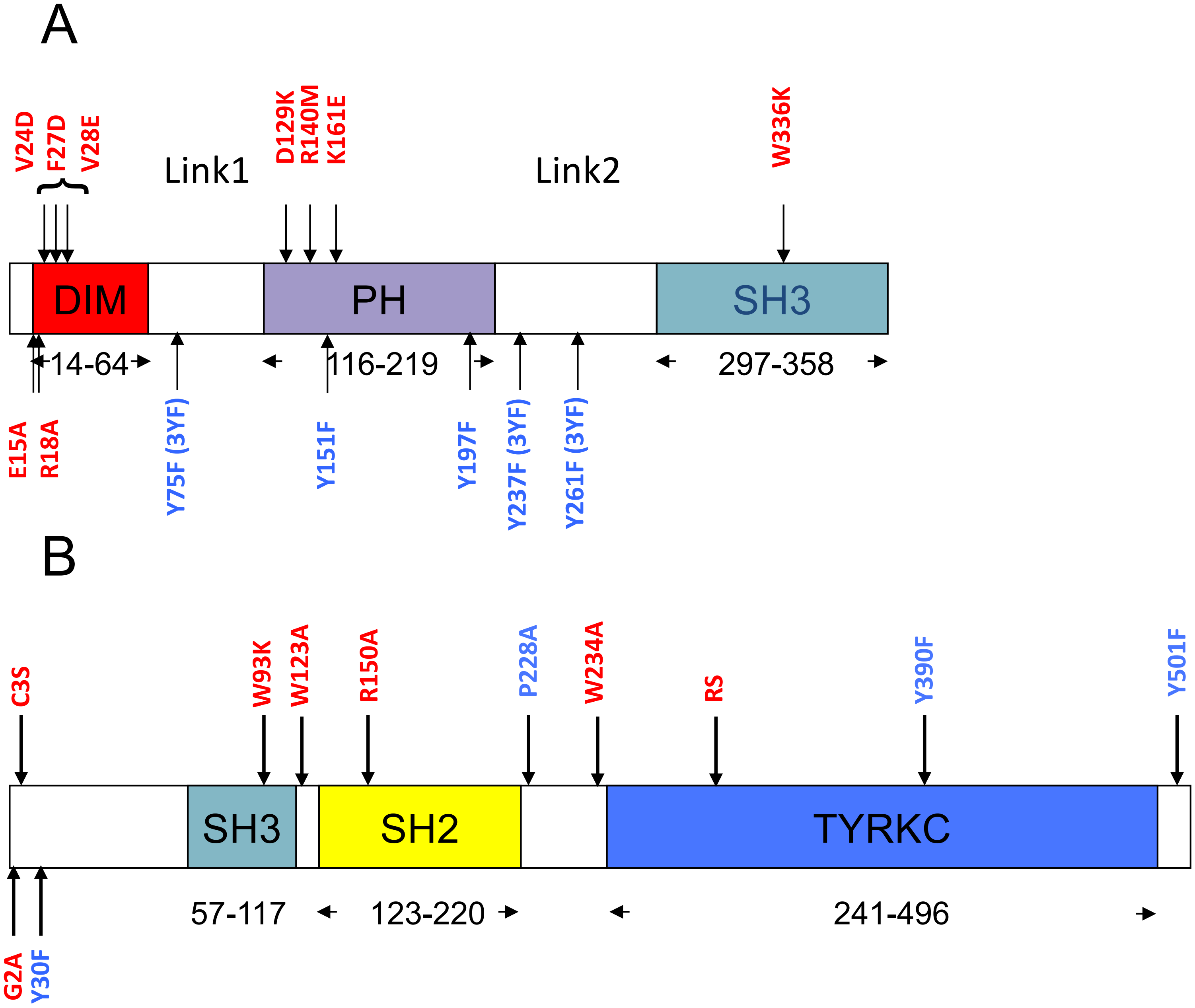

Figure S2: Conservation of the DIM domain of SKAP1 and SKAP2.

The N-terminal fragment of SKAP1 and SKAP2 protein structures were predicted from I-TASSER server. ConSurf server was used to visualize evolutionary conservation in these 2 macromolecules from their respective predict structure and aligned sequences from Clustalw program of respectively 37 and 38 protein sequences from Coelacanth to mammals. Structure was visualized using UCSF Chimera. Amino acid conservation is color-graded (1-9) from Variable (dark blue) to Conserved (dark red) through Average (white).

SKAP1 SKAP2

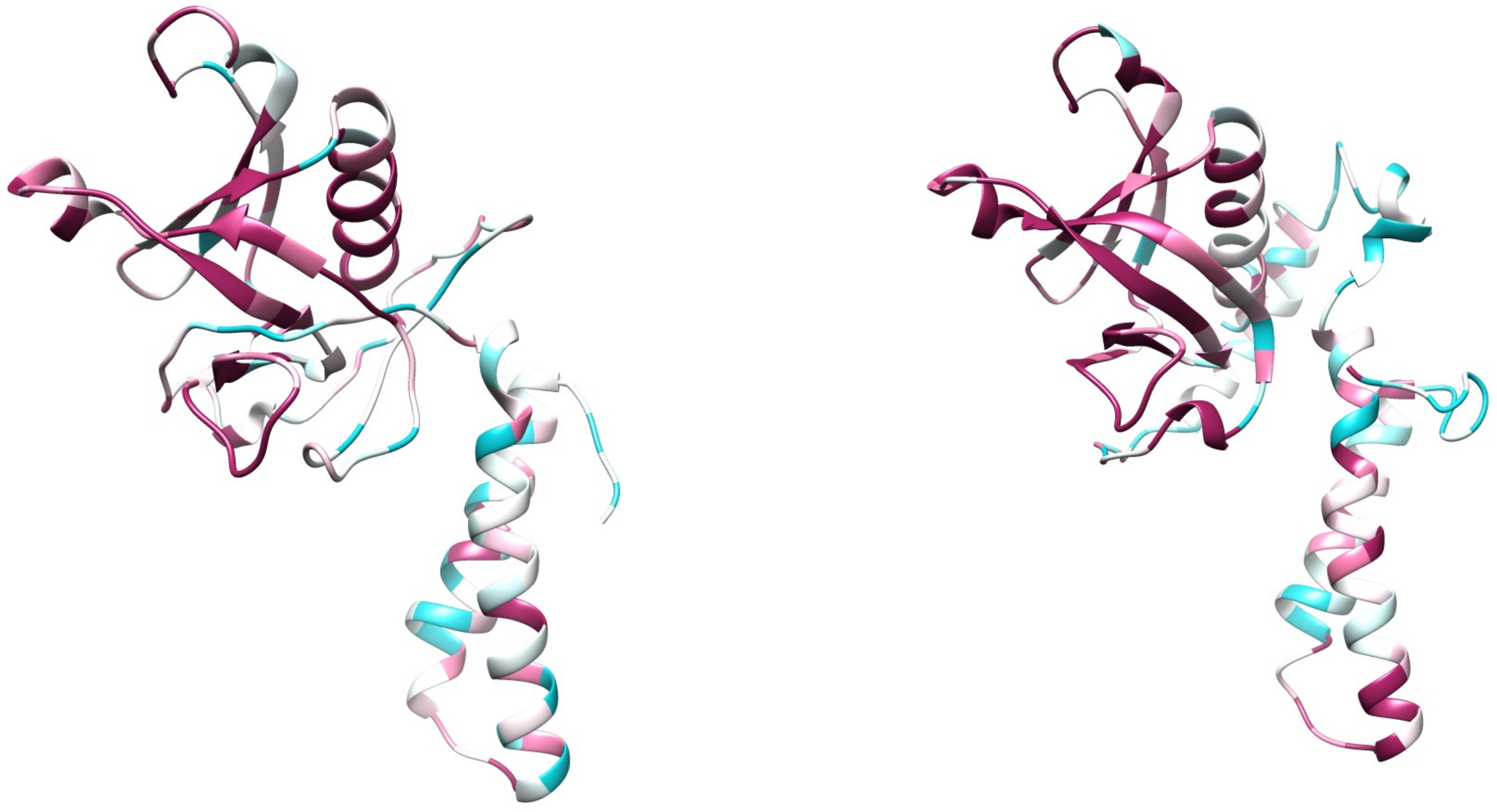

Figure S3: Effect of SKAP2 domain deletion on the interaction with SRC kinases.

Differential interaction scatterplot of SKAP2 mutant (y-axis) versus SKAP2 wild-type (x-axis) using NLR are displayed in (A-D). Interactions not affected by the mutation are aligned on the diagonal. PPI disruptive mutations are located in the lower right-hand quadrant in contrast to mutations stabilizing interaction that are located on the upper left-hand one. Interactions for N1-fused SRC kinase are in blue circle and those for C1-fused SRC kinase are in red square. Error bar: Standard Error to the Mean (SEM). A robust linear regression, which takes account of outliers, was performed on data using mmregress Stata module. (A) Scatterplot comparing the interactome of SKAP2 and DIMPH mutant. Linear regression equation is log10(DIMPH) = 0.637 * log10(SKAP2) – 0.490 for N1-fused SRC kinases. (B) Scatterplot comparing the interactome of SKAP2 and DomDIM2 mutant. Linear regression equation is log10(DomDIM2) = 0.876 * log10(SKAP2) – 0.935 for N1-fused SRC kinases. (C) Scatterplot comparing the interactome of SKAP2 and DSH3 mutant. . (D) Scatterplot comparing the interactome of SKAP2 and DPH mutant. Linear regression equations are log10(DPH) = 0.713 * log10(SKAP2) + 0.290 for N1-fused SRC kinases and log10(DPH) = 1.161 * log10(SKAP2) – 0.493 for C1-fused SRC kinases.

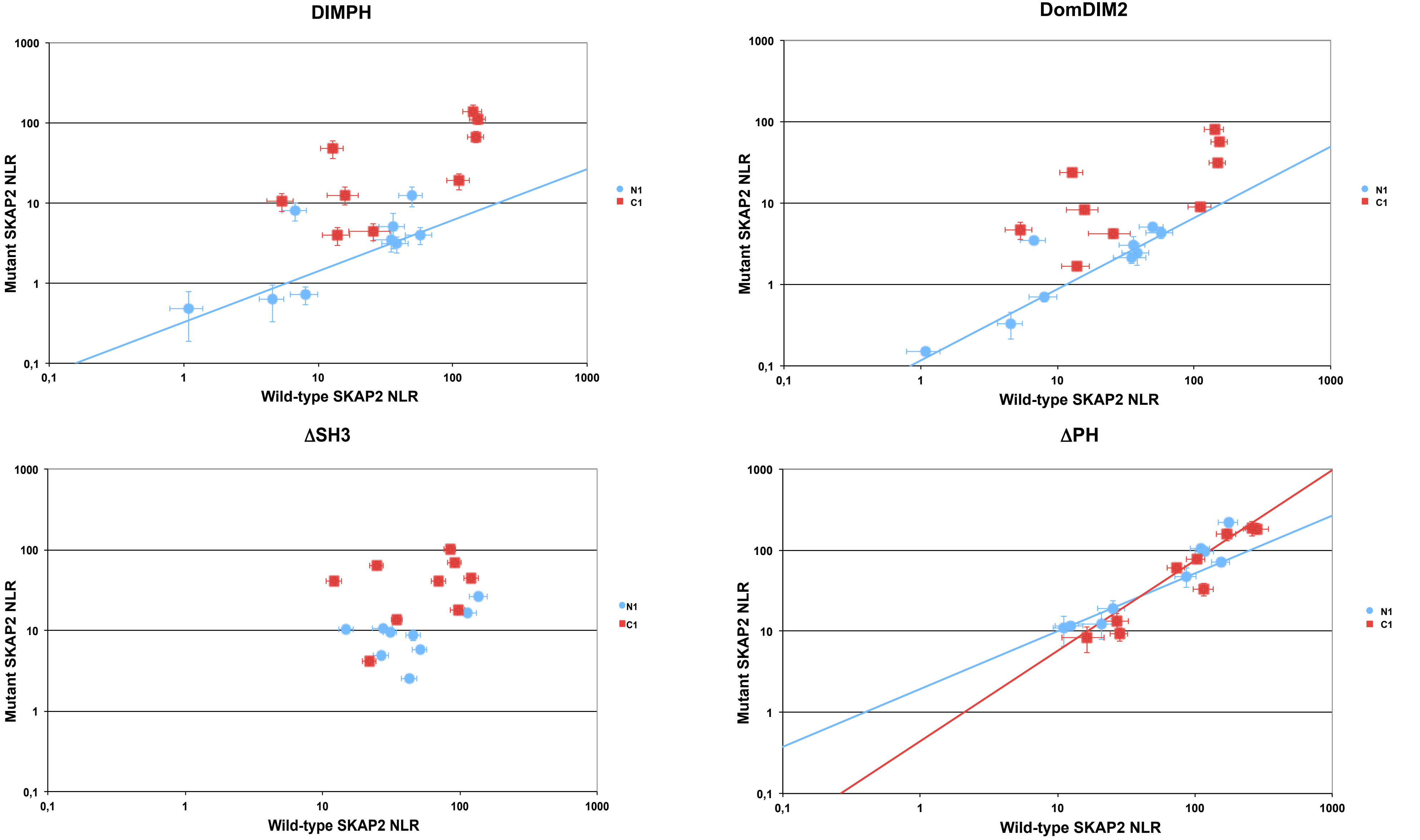

Figure S4: Effect of SKAP2 point mutations on the interaction with SRC kinase.

(A) Scatterplot comparing the interactome of SKAP2 and D129K mutant. Linear regression equations are log10(D129K) = 0.844 * log10(SKAP2) + 0.344 for N1-fused SRC kinases and log10(D129K) = 0.807 * log10(SKAP2) + 0.713 for C1-fused SRC kinases. (B) Scatterplot comparing the interactome of SKAP2 and D129K-3YF mutant. Linear regression equations are log10(D129K-3YF) = 0.887 * log10(SKAP2) + 0.065 for N1-fused SRC kinases and log10(D129K-3YF) = 0.712 * log10(SKAP2) + 0.745 for C1-fused SRC kinases. (C) Scatterplot comparing the interactome of SKAP2 and 3YF mutant. Linear regression equations are log10(3YF) = 0.669 * log10(SKAP2) + 0.330 for N1-fused SRC kinases and log10(3YF) = 0.946 * log10(SKAP2) + 0.005 for C1-fused SRC kinases. (D) Scatterplot comparing the interactome of SKAP2 and R140M mutant. Linear regression equations are log10(R140M) = 1.067 * log10(SKAP2) – 0.147 for N1-fused SRC kinases and log10(R140M) = 1.392 * log10(SKAP2) – 0.969 for C1-fused SRC kinases. (E) Scatterplot comparing the interactome of SKAP2 and DIM$^-$ mutant. Linear regression equations are log10(DIM$^-$) = 0.716 * log10(SKAP2) + 0.020 for N1-fused SRC kinases and log10(DIM$^-$) = 1.435 * log10(SKAP2) – 1.262 for C1-fused SRC kinases. (F) Scatterplot comparing the interactome of SKAP2 and SKAP1 proteins. Linear regression equations are log10(SKAP1) = 0.749 * log10(SKAP2) + 0.453 for N1-fused SRC kinases and log10(SKAP1) = 0.790 * log10(SKAP2) + 0.116 for C1-fused SRC kinases.

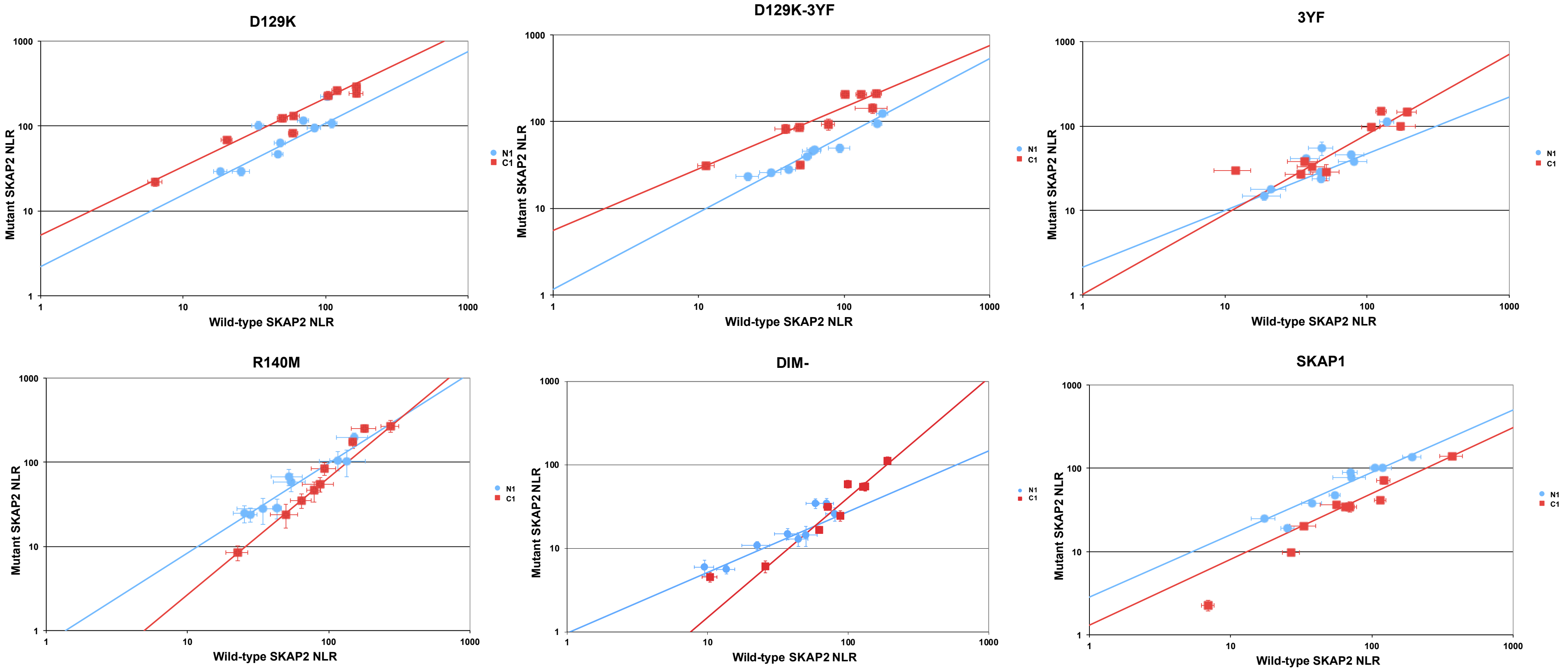

Figure S5: Impact of HCK mutations on SKAP2 interactions.

In PPI-mutation plot, Y-axis represents NLR of each HCK mutant in a logarithmic scale and X-axis, the position of the mutation. NLR are normalized according to that of HCK-N1. (A) The upper panel shows the PPI-mutation plot of 20 HCK mutants from 11 mutations and the lower panel a schematic of HCK organization. (B) Similar data than (A) after zooming to the higher values.

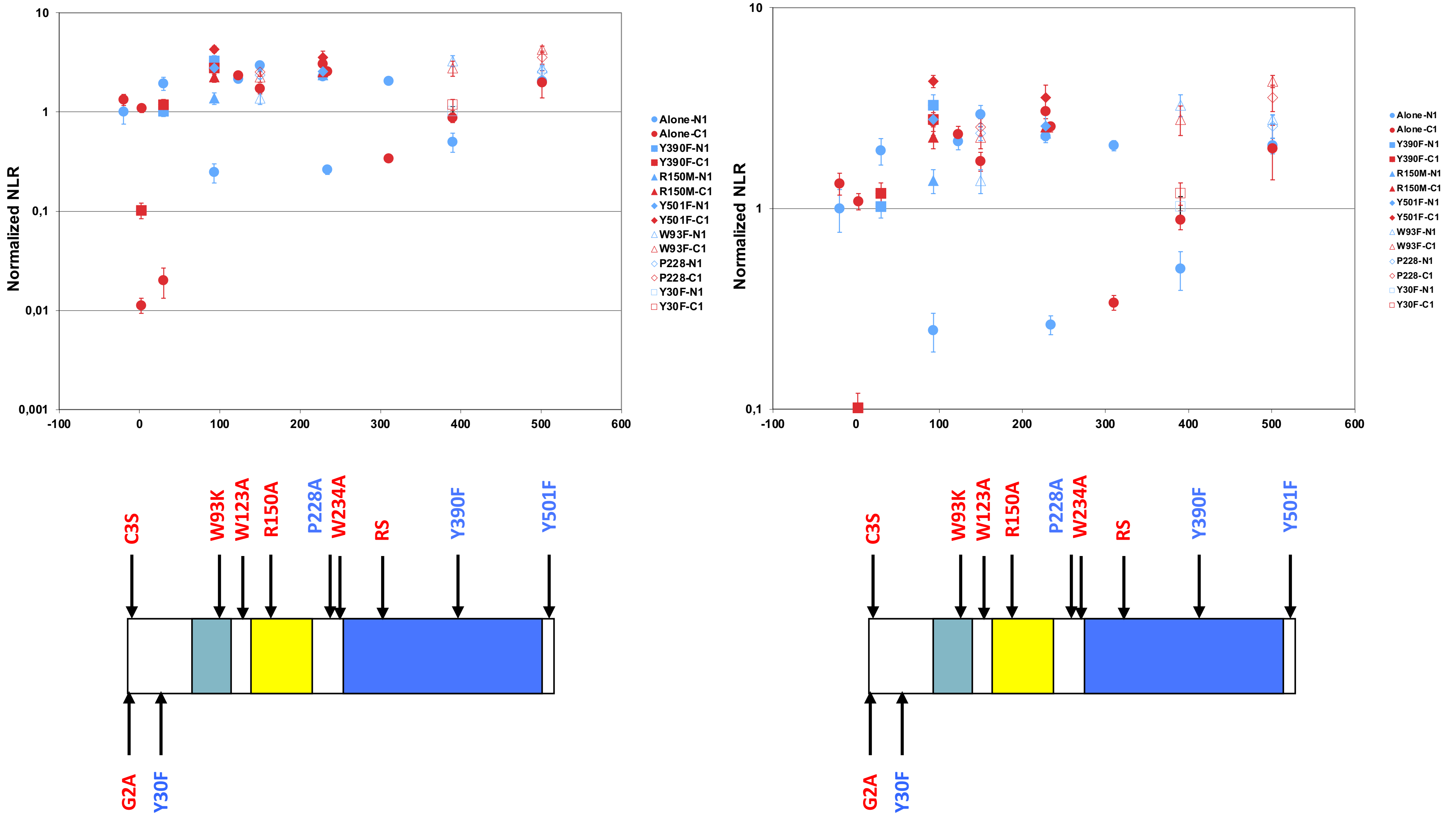

Figure S6: NLR means of N1 or C1-fused wild-type SRC kinases. Data of the 31 experiments with SKAP2 mutants were used to calculate for each of the nine wild-type SRC kinases either N1-fused (red) or C1-fused (blue) the mean of the NLR ± SEM.

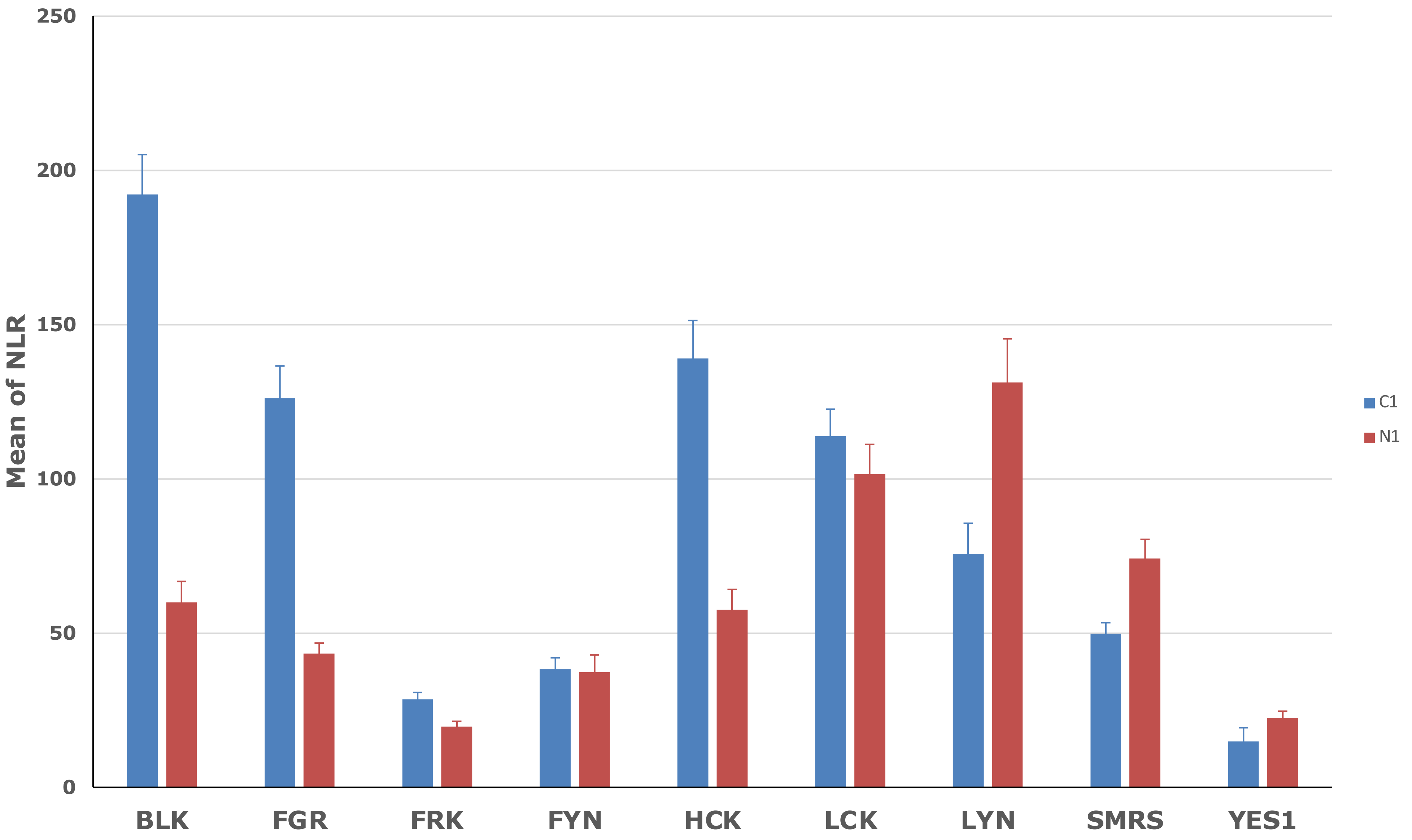

Figure S7: Role of Y390F and K7R mutation of protein stability of five HCK mutants.

In PPI-mutation plot, Y-axis represents NLR of each HCK mutant in a logarithmic scale and X-axis, the position of the mutation. NLR are normalized according to that of HCK-N1. (A) The upper panel shows the PPI-mutation plot of experiment of panel (A) of Figure 6 and the lower panel is a schematic of HCK organization. (B) The upper panel shows the PPI-mutation plot of experiment of panel (B) of Figure 6 and the lower panel is a schematic of HCK organization.

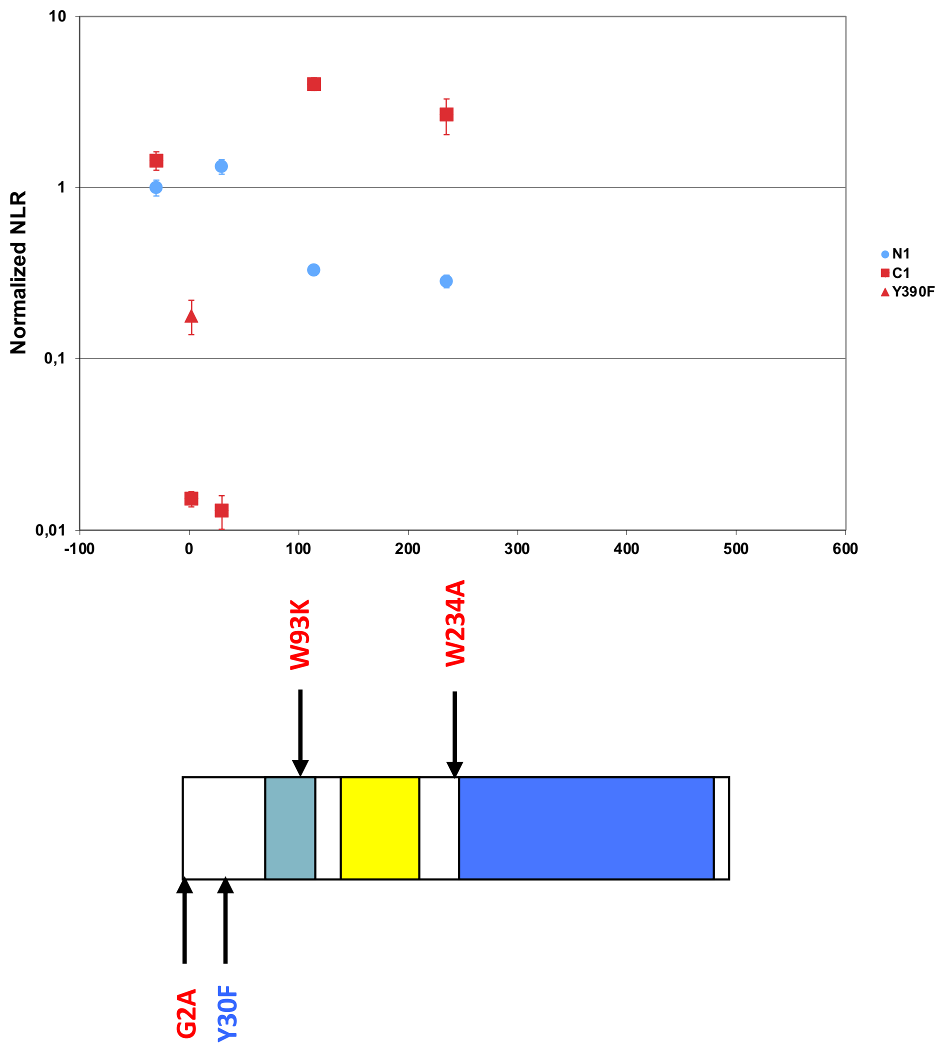

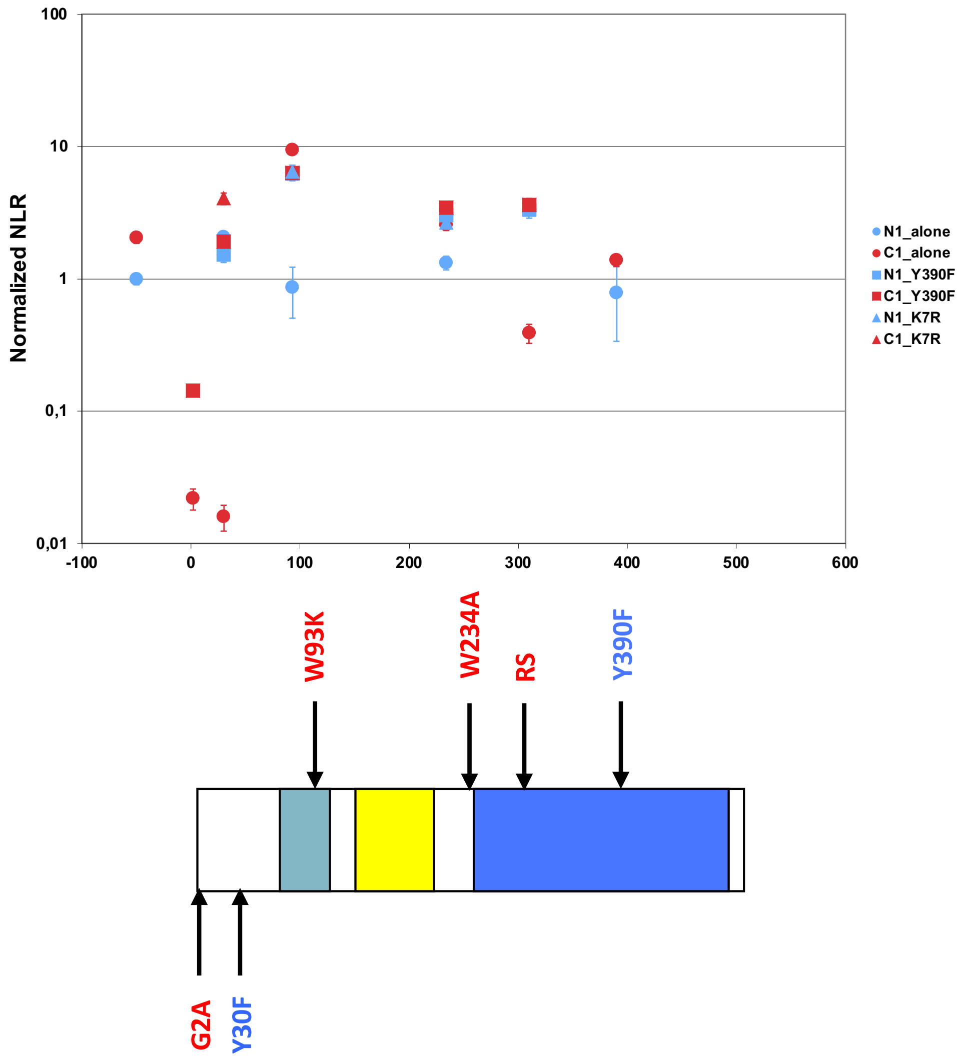

Supplement: Supplemental Figures S1–S7 [file mmc2.pdf]
